# Subventricular zone neural progenitors reverse TNF-alpha effects in cortical neurons

**DOI:** 10.1186/s13287-015-0158-2

**Published:** 2015-09-07

**Authors:** Raffaella Morini, Elsa Ghirardini, Erica Butti, Claudia Verderio, Gianvito Martino, Michela Matteoli

**Affiliations:** Department of Medical Biotechnology and Traslational Medicine, University of Milano, via Vanvitelli 32, 20129 Milan, Italy; Humanitas Clinical and Research Center, via Manzoni 56, 20089 Rozzano, Italy; Institute of Experimental Neurology, Division of Neuroscience, San Raffaele Scientific Institute and University, Via Olgettina 58, 20132 Milan, Italy; National Research Council, Institute of Neuroscience, via Vanvitelli 32, 20129 Milan, Italy

## Abstract

**Introduction:**

Tumor necrosis factor alpha (TNFα) plays a physiological role in controlling synaptic transmission and plasticity in the healthy central nervous system by modulating glutamate receptor trafficking to the plasma membrane. TNFα expression is also rapidly induced in response to tissue injury and infection. By promoting the insertion of Ca^2+^ permeable-AMPA receptors into the neuronal plasma membrane, this cytokine may cause excessive Ca^2+^ influx into neurons, thus enhancing neuronal death.

**Methods:**

Primary cultures of cortical neurons were obtained from E18 foetal mice and incubated for 24 h with adult neural stem cells (aNPCs) either stimulated with lipopolysaccharide (LPS^+^aNPCs) or not (aNPCs). Cultures were treated with TNFα (100 ng/ml), and electrophysiological recordings were performed in different conditions to evaluate the effect of the cytokine on neuronal transmission.

**Results:**

In this study, we demonstrate that aNPCs from the subventricular zone reverse the effects induced by the cytokine. Moreover, we show that the effect of aNPCs on cortical neurons is mediated by cannabinoid CB1 receptor activation.

**Conclusion:**

These data suggest that the role of aNPCs in preventing excitatory neurotransmission potentiation induced by TNFα on cortical neurons may have important implications for pathologies characterized by an inflammatory component affecting cortical neurons such as Alzheimer’s disease.

**Electronic supplementary material:**

The online version of this article (doi:10.1186/s13287-015-0158-2) contains supplementary material, which is available to authorized users.

## Introduction

The pro-inflammatory cytokine tumor necrosis factor alpha (TNFα), released by glial cells, is involved in several brain functions. Under physiological conditions, the cytokine is thought to be responsible for the activity-dependent homeostatic regulation of synaptic connectivity, which consists of the increase of synaptic strength in response to prolonged blockade of activity. This process, termed synaptic scaling, involves the insertion of AMPA receptors, mobilized from intracellular stores, into the plasma membrane [1–4].

However, because an excess of cell surface localized AMPA receptors might predispose the neuron to glutamate-mediated excitotoxicity and excessive intracellular calcium concentrations, an increase in the amount of TNFα released by microglia as a consequence of immune activation may lead to neuronal death [5, 6]. In recent years, TNFα actions have been implicated consistently and increasingly in central nervous system (CNS) pathology. In particular, elevated TNFα levels have been reported in multiple sclerosis, stroke, ischemia, and epilepsy [7–12]. A crucial role of TNFα has also been reported in Alzheimer’s disease (AD) [13]. More specifically, TNFα is upregulated in patients with AD [14], and polymorphisms of the TNFα gene affect the risk of developing AD [15].

Recent evidence has indicated that microglial TNFα production plays a critical role in the induction of neuronal cell cycle events in AD, which are toxic for terminally differentiated neurons and lead to neurodegeneration [16]. Consistent with a role of TNFα in AD, perispinal administration of etanercept, a potent anti-TNF fusion protein, produced sustained clinical improvement in a 6-month, open-label pilot study in patients with mild to severe AD (reviewed in [17]).

It has recently been found that adult neural stem/precursor cells (aNPCs) located within the subventricular zone (SVZ) adapt their functions to the needs of the tissue, carrying out a broad spectrum of “bystander” non-neurogenic roles aimed at the maintenance of brain homeostasis, besides purely neurogenic functions [18]. In chronic inflammatory conditions, NPCs seem to be driven toward cell replacement, differentiating into new cellular elements in order to replace lost endogenous cells. Conversely, in acute inflammatory conditions, NPCs tend to remain undifferentiated and promote CNS tissue healing via the secretion of immunomodulatory and neuroprotective molecules that are capable of reducing detrimental tissue responses (reviewed in [19]). A continuous crosstalk between immune cells and NPCs appears to determine both the efficacy of endogenous regenerative responses and mechanism of action as well as the fate and functional integration of grafted NPCs, through different kinds of receptors located on their membranes [20, 21].

The non-neurogenic function has been demonstrated in stroke, where newly formed NPCs that were not integrated into the neural network were shown to provide protection from tissue injury via the secretion of neurotrophic factors [22, 23]. Additionally, NPCs have been recently found to play an “innate” homeostatic regulatory role, likely aimed at the prevention of reactive inflammation, by protecting striatal neurons from glutamate-mediated excitotoxicity [24]. More specifically, SVZ-derived aNPCs reverted the increased frequency and duration of spontaneous excitatory post-synaptic currents induced by exposure of striatal neurons to TNFα [24]. Whether the protective effect of aNPCs on striatal neurons extends to different neuronal populations subjected to pathological insults remains unknown. Given the established pathological role of TNFα in different diseases, we aimed at investigating whether SVZ-derived aNPCs are able to revert the effects of this cytokine also in cortical neurons, which represent the main target cells for neurodegenerative processes such as those occurring in Alzheimer’s disease.

## Methods

### Ethics statement

All the experimental procedures followed the guidelines established by the Italian Council on Animal Care and were approved by Italian Government decree 27/2010.

### Cell cultures and drug treatments

Primary neuronal cultures from cerebral cortex were obtained from E18 C57Bl/6 mice as described by Bartlett and Banker (1984) [25] with slight modifications as in [26]. Briefly, cortices were removed and dissociated by treatment with trypsin (0.125 % for 15 min at 37 °C) followed by trituration with a polished Pasteur pipette. The dissociated cells were plated onto glass coverslips coated with poly-*L*-lysine at a density of 400 cells/mm^2^. The cells were maintained in Neurobasal medium (Waltham, MA USA 02451) with B27 supplement, penicillin-streptomycin (10,000 units/ml), 2 mM glutamine, and 12.5 μM glutamate (neuronal medium).

TNFα (100 ng/ml) was incubated with neurons for 30 min prior to electrophysiological recordings and was present during the recordings [2], in the presence or absence of the neutralizing mouse TNF-α antibody (0.5 μg/ml; R&D Systems, Minneapolis, MN, USA) to neutralize the biological activity of mouse TNF-α. The cannabinoid antagonist AM251 was added to the cortical neurons at a final concentration of 5 μM for 2 h and was present during recordings [27].

### Generation and maintenance of aNPC cultures

aNPC cultures were obtained from 6-week-old C57Bl/6 mice, as previously described [28]. Briefly, 3-mm-thick coronal sections were obtained from the anterior forebrain of 6-week-old mice (2 mm from the anterior pole of the brain). Dorsal SVZs were carefully dissected by using fine scissors in the following dissociating medium: Earl’s Balanced Salt Solution (Gibco, Invitrogen) supplemented with 1 mg/ml Papain 27 U/mg (Sigma-Aldrich, St. Louis, MO, USA), 0.2 mg/ml Cysteine (Sigma-Aldrich), and 0.2 mg/ml EDTA (Sigma-Aldrich). Then, dissected tissue was incubated in the same solution for 30 min at 37 °C on a rocking platform. Finally, dissociated cells were plated in standard neurosphere growth medium Neurocult proliferation medium (Stemcell Technology, Vancouver, BC, Canada) supplemented with epidermal growth factor (EGF) (20 ng/ml) and fibroblast growth factor 2 (FGF2) (10 ng/ml). For each *in vitro* passage, single cells were obtained by incubating neurospheres in Accumax (Sigma-Aldrich) for 10 min, and then 8000 cells/mm^2^ were plated on T75 plastic flasks (Nunc, Rochester, NY, USA). Neurospheres were propagated *in vitro* and assayed for self-renewal and cell differentiation after six passages, as previously described [28].

To label aNPCs, cells were transduced with a lentivirus [29] expressing green fluorescent protein (GFP); about 1.5 × 10^6^ cells in 10 ml were infected with 3 × 10^6^ tu/ml of GFP-lentivirus. The day after the transduction, the medium was replaced with a fresh one and cells were allowed to grow. For LPS stimulation, aNPCs were dissociated, incubated with 100 ng/ml of LPS (026:B6; Sigma-Aldrich), and then collected after 16 h.

### Cell culture electrophysiology

Whole-cell voltage-clamp recordings were performed on mouse primary cortical neurons at 13–15 days *in vitro*. During recordings, cells were bathed in a standard external solution containing (in mM): 125 NaCl, 5 KCl, 1.2 MgSO_4_, 1.2 KH_2_PO_4_, 2 CaCl_2_, 6 glucose, and 25 HEPES-NaOH, pH 7.4. Recording pipettes were fabricated from borosilicate glass capillary by using a horizontal puller (Sutter Instrument Company, Novato, CA, USA) inducing tip resistances of 3–5 MΩ and filled with a standard intracellular solution containing (in mM): 130 K-gluconate, 10 KCl,1 EGTA, 10 HEPES- NaOH, 2 MgCl_2_, 4 MgATP, and 0.3 Tris-GTP. For miniature excitatory post-synaptic current (mEPSC) recordings, 1 μM tetrodotoxin, 20 μM Bicuculline, and 50 μM AP5 (Tocris Bioscience, Bristol, UK) were added to standard extracellular solution to block spontaneous action potential propagation, GABA-A, and NMDA receptors, respectively. Recordings were performed at room temperature in voltage clamp mode at a holding potential of −70 mV by using a Multiclamp 700B amplifier (Molecular Devices, Sunnyvale, CA, USA) and pClamp-10 software (Axon Instruments, Foster City, CA, USA). Series resistance ranged from 10 to 20 MΩ and was monitored for consistency during recordings. Cells in culture with leak currents of more than 100 pA were excluded from the analysis. Signals were amplified, sampled at 10 kHz, filtered to 2 or 3 KHz, and analyzed by using pClamp 10 data acquisition and analysis program. To verify the specific effect of adult NPCs, cultured cortical neurons were incubated for 24 h prior to electrophysiological recordings with either lipopolysaccharide-stimulated (LPS^+^aNPCs) or unstimulated aNPCs. Only cortical neurons surrounded by aNPCs were selected to record.

### Immunofluorescence staining of dissociated neurons

Neuronal cultures were fixed with 4 % paraformaldehyde and 4 % sucrose. The following antibodies were used: rabbit anti-CB1 (1:500; Synaptic System, Goettingen, Germany), guinea pig anti-vGLUT1 (1:1000; Synaptic System), and guinea pig anti -VGAT (1:750; Synaptic System). Secondary antibodies were conjugated with Alexa-488 and Alexa-555 fluorophores (Invitrogen).

Images were acquired by using a Leica Spe confocal microscope equipped with an ACS APO 63.0X1.3 objective (Leica, Wetzlar, Germany).

Pixel size was 94.8 nm_94.8 nm, and acquisition parameters (i.e., laser power, gain, and offset) were kept constant across different experimental settings. The minimum puncta size was set at three pixels.

Colocalization of two selected markers was measured by using the boolean function “and” for the selected channels. The resulting image was binarized, inverted, and used as a colocalization mask to be subtracted from single channel. The number of puncta resulting from colocalization mask subtraction (colocalizing puncta) was measured for each marker. A colocalization ratio was set as colocalized area/total puncta aerea. Fluorescence image processing and analyses were performed with the ImageJ Software (National Institutes of Health, Bethesda, MD, USA).

### Hoechst 33342/PI double-stain cell death detection essay

To distinguish between living and dead cell populations and assess the percentage of neuronal death after AM251, cultured neurons were incubated for 15 min at 37 °C with Hoechst 33342 (final concentration, 1 ug/ml; Sigma-Aldrich) and propidium iodide (PI) (final concentration, 1 μg/ml; Sigma-Aldrich) dissolved in neuronal medium. PI is a red-fluorescence dye (excitation/emission maxima ~535/617 nm, when bound to DNA) which is only permeant to dead cells, whereas the blue-fluorescent Hoechst 33342 dye (excitation/emission maxima ~350/461 nm, when bound to DNA) is specifically used to stain the nuclei of living or fixed cells and tissues. Fluorescence images resulting from the simultaneous use of these dyes, acquired by using an inverted Olympus IXB53 microscope (Olympus, Tokyo, Japan), allowed us to determine the number of dead neurons in our experimental conditions.

### Statistical analysis

Patch clamp experiments were analyzed by using pClamp 10 data acquisition and analysis program and fluorescence image processing and analyses were performed with the ImageJ Software. Statistical analysis was performed by using PRISM 6 software (GraphPad Software Inc., San Diego, CA, USA). After testing whether data were normally distributed or not, the appropriate statistical test has been used (see figure legends). n refers to the number of elements analysed. Data are presented as mean ± standard error of the mean from the indicated number of elements analysed. The differences were considered to be significant if *P* value of less was than 0.05 and are indicated by an asterisk; those at *P* value of less than 0.01 are indicated by double asterisks.

## Results

### TNFα increases amplitude and frequency of miniature currents in primary cortical neurons

**Fig. 1:**
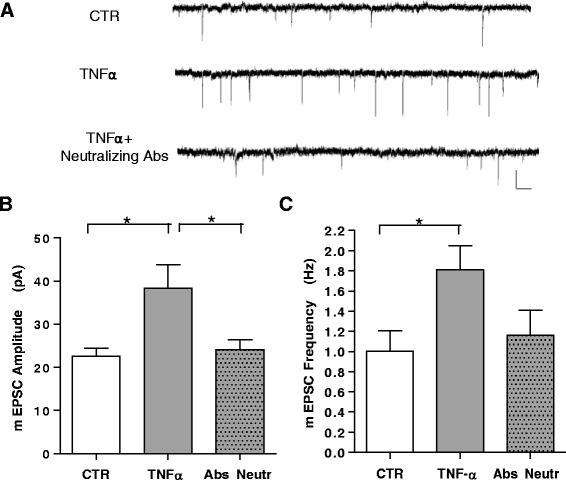
TNFα increases mEPSC frequency and amplitude in cortical neurons. **a** Examples of voltage-clamp recordings of mEPSCs from cultured cortical neurons in control conditions, upon treatment with TNFα (100 ng/ml 30 min) or in the presence of TNFα and neutralizing Abs (0.5 μg/ml; R&D Systems) (calibration bars: 20 pA, 200 ms). **b, c** Group data of average mEPSC amplitude and frequency in untreated (n = 15), TNFα-treated (n = 13), and TNFα plus neutralizing Ab-treated (n = 10) cells. A significant increase in average mEPSC amplitude and frequency is produced by TNFα, completely blocked by neutralizing anti -TNFα Abs (*P* < 0.05). All data are expressed as mean ± standard error of the mean. Statistical test: one-way analysis of variance, Dunn’s multiple comparisons test. *Ab* antibody, *mEPSC* miniature excitatory post-synaptic current, *TNFα* tumor necrosis factor alpha

Although several studies have shown that TNFα increases synaptic strength at hippocampal excitatory synapses by rapidly inserting AMPA receptors via TNFR1 receptor activation [30], little has been reported in different neuronal cell types [31, 32]. To address whether this effect is restricted to hippocampal neurons or also occurs at cortical synapses, we grew cultures of primary neurons derived from the cerebral cortex and exposed them to TNFα (100 ng/ml) for 30 min before recording mEPSCs. Our data showed a significant increase in both frequency and amplitude of mEPSCs in TNFα-treated neurons with respect to untreated control neurons (Fig. 1). This effect was completely blocked in the presence of neutralizing TNFα antibodies. These data indicate that cortical neurons respond to TNFα in a manner that is similar to hippocampal neurons and indicate that this cytokine may predispose cortical neurons to excitotoxic phenomena.

### Adult NPCs revert the effect of TNFα treatment in cortical neurons

**Fig. 2:**
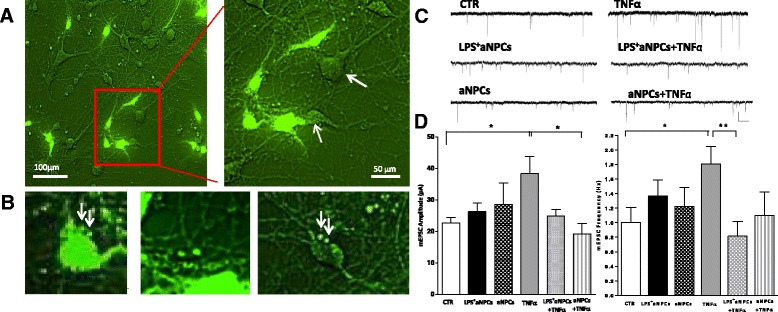
aNPCs treated with LPS prevent the increase in mEPSC frequency produced by TNFα in cortical neurons. **a** Representative fluorescence microscopy images of cortical neurons incubated for 24 h with aNPCs either lipopolysaccharide-stimulated (LPS^+^aNPCs) or not (aNPCs) (*green cells*). The *right panel* shows higher magnification (scale bar: 100 μm; 50 μm). Only cortical neurons surrounded by aNPCs were selected to record (*white arrows*). **b** Details of (a) show GFP-positive membranous structures of small and medium size resembling round membrane vesicles. **c** Representative traces of voltage-clamp recordings of mEPSCs from cultured cortical neurons in control conditions or from neurons treated with LPS^+^aNPCs/aNPCs, TNFα, or LPS^+^aNPCs/aNPCs with TNFα (100 ng/ml 30 min) (calibration bars: 20 pA, 200 ms). **d** Group data of average mEPSC amplitude and frequency of control (n = 15), LPS^+^aNPCs-treated (n = 12), aNPCs-untreated (n = 8), TNFα-treated (n = 13), LPS^+^aNPC and TNFα-treated (n = 10), and aNPC-untreated and TNFα-treated (n = 8) cells. The increase in mEPSC frequency due to TNFα is completely reverted by LPS^+^aNPCs (*P* < 0.05). The increase in mEPSC amplitude due to TNFα is completely reverted by aNPC treatment (*P* < 0.05). A clear, though not significant, tendency toward reduction is detected in mEPSC amplitude or frequency in neuronal cultures exposed to LPS^+^aNPCs or aNPCs, respectively. All data are expressed as mean ± standard error of the mean. Statistical test: one-way analysis of variance, Dunn’s multiple comparisons test. *aNPC* adult neural stem cell, *GFP* green fluorescent protein, *LPS* lipopolysaccharide, *LPS* ^*+*^ *aNPC* lipopolysaccharide-stimulated adult neural stem cell, *mEPSC* miniature excitatory post-synaptic current, *TNFα* tumor necrosis factor alpha

Both injury and neurodegenerative diseases increase the amount of TNFα in the brain and this contributes to neuronal death. Recent studies demonstrated that SVZ-derived aNPCs protect striatal neurons from glutamate-mediated toxicity in pathological conditions associated with inflammation and excitotoxicity, such as epilepsy and ischemic stroke [24]. To assess whether the neuroprotective effects of aNPCs extend to different neuronal types, we exposed neurons to TNFα before or after incubating them for 24 h with LPS^+^aNPCs in order to mimic the effects of different danger (or recognition) signals triggering the inflammatory response. aNPCs were infected in order to express GFP [29], and only neurons surrounded by aNPCs were selected to record (Fig. 2a). Notably, membranous structures of small and medium size, including round membrane vesicles, were detected on the cell surface of aNPCs (Fig. 2b left panel), as recently described [33]. These vesicles were released by aNPCs (Fig. 2b middle panel) and adhered to GFP-negative neuronal cells (Fig. 2b right panel).

Voltage clamp recordings of cortical neurons surrounded by aNPCs revealed that the increase in average mEPSC frequency due to TNFα was fully prevented by treatment of the cultures with LPS^+^aNPCs (Fig. 2). A clear tendency for recovery of mEPSC amplitude was also observed, although statistical significance was not reached. No difference in mEPSC frequency and amplitude was detected when LPS^+^aNPCs were added to cell cultures in the absence of TNFα (Fig. 2). Furthermore, compared with striatal neurons where aNPCs exert a regulatory role only when previously exposed to LPS [24], both LPS-treated and LPS-untreated NPCs appeared effective in preventing the changes in mEPSC frequency and amplitude in cortical neurons following TNFα administration. However, in the case of LPS-untreated aNPCs, a statistically significant recovery was evident only in mEPSC amplitude (Fig. 2). These data indicate that aNPCs can revert the TNFα-induced mEPSC alterations.

### Adult neural stem/progenitor cells act via CB1 receptor activation

**Fig. 3:**
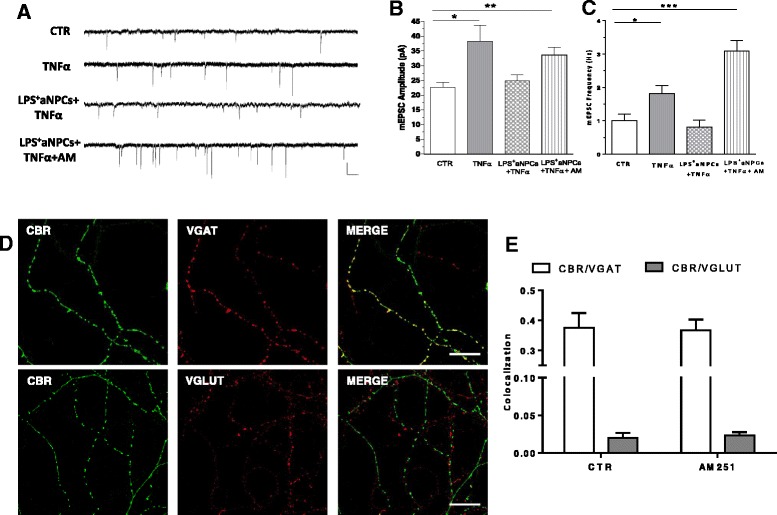
The CB1 antagonist AM251 blocks the rescue of mEPSC changes sustained by LPS^+^aNPCs in TNFα-treated cultures. **a** Representative electrophysiological traces of mEPSCs under the different treatment conditions. **b, c** Group data of average mEPSC amplitude and frequency of untreated (n = 15), TNFα-treated (n = 13), LPS^+^aNPCs + TNFα (n = 10), and LPS^+^aNPCs + TNFα + AM 251-treated (n = 9) cells. LPS^+^aNPC treatment fails to revert TNF effect in the presence of AM 251 (*P* < 0.05, *P <* 0.01). **d** Confocal microscopy images showing expression of CB1 receptor in cultured cortical neurons. Immunofluorescence for CB1 (*green*), VGAT (*red*), and Merge in the *upper panels* and for CB1 (*green*), VGLUT (*red*) and Merge in *lower panels* is shown. Scale bars, 20 μm. **e** Summary bar graphs showing the colocalization of CB1 and VGAT or VGLUT. All data are expressed as mean ± standard error of the mean. Statistical test: one-way analysis of variance, Dunn’s multiple comparisons test. *CB1* type 1 cannabinoid receptor, *LPS* ^*+*^ *aNPC* lipopolysaccharide-stimulated adult neural stem cell, *mEPSC* miniature excitatory post-synaptic current, *TNFα* tumor necrosis factor alpha

The protective role of type 1 cannabinoid receptors (CB1), against enhanced excitotoxic death caused by TNFα and other harmful agents, has been demonstrated by several studies [34, 35]. Furthermore, it has been found that aNPCs are capable of secreting endogenous cannabinoids [24, 36]. We therefore aimed to test whether, in our experimental conditions, aNPCs exert their described effect through CB1 receptor activation. To this end, we recorded mEPSCs in TNFα-treated cortical neurons incubated with aNPCs in the presence of the CB1 antagonist AM251 (Fig. 3). The results showed that LPS^+^aNPC treatment fails to revert TNFα effects in the presence of AM251, indicating that aNPCs act through CB1 receptor activation. As already shown in previous reports [37–39], CB1 receptors were expressed mainly in inhibitory neurons (Fig. 3d) and their localization did not change when neurons were exposed to AM251 (Fig. 3e). Our results demonstrate that CB1 is present in both inhibitory and excitatory synapses but that the signal overlap is approximately 10 times higher in inhibitory synapses than in excitatory ones (Fig. 3e). Additional file 1: Figure S1 shows that AM251 *per se* did not significantly change mEPSC frequency and amplitude (A), did not display cytotoxic effects (B), and did not change the synaptic expression of CB1 receptors (C, D).

## Discussion

TNFα plays a physiological role in controlling synaptic transmission and plasticity in the healthy CNS by modulating glutamate receptor trafficking to the plasma membrane [2, 3]. Excitatory synapse scaling, caused by blockade of neuronal activity, is known to be mediated by TNFα which increases the insertion of AMPA receptors (of both GluR1 and GluR2 subunits) into the plasma membrane and decreases GABA-A receptor cell surface expression. Through this process, TNFα increases the mean frequency and amplitude of mEPSCs [31, 40, 41]. Consistently, in hippocampal cultures from TNFα-deficient mice, no increase in mEPSC amplitude resulted from activity blockade, thus confirming the TNFα dependence of this increase [4]. In the present study, we confirm the effects of TNFα in mEPSCs recorded from primary cultures of cortical neurons.

TNFα is rapidly induced in the CNS in response to tissue injury and infection [42, 43]. Although astrocytes and neurons are able to produce TNFα, microglial cells are believed to be the major source of this cytokine during neuroinflammation [44]. Through TNFR1 signaling, TNFα promotes further cytokine release from microglia, as well as increasing glutamate exocytosis and inhibiting glutamate uptake from astrocytes, thus elevating extracellular glutamate levels. Under such conditions, the TNFα-mediated insertion of Ca2^+^ permeable-AMPA receptors into the neuronal plasma membrane may cause excessive Ca2^+^ influx into neurons and induce neuronal death. The dying neurons maintain microglia in an active state, releasing TNFα. Through this loop, TNFα provides a link between neuroinflammatory and excitotoxic processes (reviewed in [45]).

It has recently been shown that SVZ-derived aNPCs play a role in protecting striatal medium spiny neurons from glutamatergic excitotoxicity, by reverting the increased frequency and duration of spontaneous excitatory post-synaptic currents induced by toxic or ischemic stressful stimuli or both [24]. In the present study, we demonstrate that aNPCs revert the effect of TNFα treatment also in cortical neurons, fully preventing the increase in mEPSC frequency. The increase in mEPSC amplitude, a typical consequence of TNFα treatment [2], was not observed in striatal neurons [24] where, besides frequency, only half width and decay time of mEPSCs were affected. Also, compared with previous work on striatal tissue slices [24], where the addition of LPS-exposed aNPCs, but not of untreated aNPCs, reverted the EPSC changes produced by TNFα, both untreated and LPS-treated aNPCs were effective in reverting either the frequency or the amplitude of TNFα effects in cortical neurons. These data indicate the existence of subtle, but potentially significant, differences among different brain areas in terms of both cytokine responsivity and the potential protective effects of aNPCs.

Although we cannot exclude that factors other than endocannabinoids may contribute to the observed phenomenon, we show that aNPC effects on cortical neurons are mediated by the activation of CB1 receptors, as demonstrated by the blockade on aNSC-mediated recovery of TNFα effects by the CB1 antagonist AM251. It is likely that the activation of CB1 receptors is a consequence of the secretion of endocannabinod arachidonoyl ethanolamide (AEA), a molecule that regulates glutamatergic tone through CB1 binding [24], by aNPCs. Endocannabinoids are likely to be released from aNPCs through extracellular membrane vesicles, which have been recently found to allow the movement of lipophilic endocannabinoids into the extracellular space to reach target neurons [38]. Notably, in line with Cossetti and colleagues [33], we have detected the formation of extracellular vesicles from the cell surface of aNPCs. These vesicles adhere to the membrane of cortical neurons, thus supporting the concept that they may actively mediate the communication of neural stem/precursor cells with the microenvironment. It ensues from this that stem cell therapies may work not only via cell replacement but also through a consistent intercellular molecule exchange [33].

The preventive effect of aNPCs on the potentiation of excitatory neurotransmission induced by TNFα in cortical neurons may have important implications. Indeed, across different pathological conditions, TNFα is thought to contribute to neuroinflammation in AD. Aβ_1–40_ induces an increase in TNFα expression and oxidative alterations in the prefrontal cortex and hippocampus [46]. In support of the role of TNFα as a central mediator of Aβ action, excess cytokine, at a level 25 times higher than controls, has been reported in the cerebrospinal fluid of patients with AD, which correlates with progression from mild cognitive impairment to AD [47]. Quite notably, a percentage of neurons localized near the plaques of amyloid β deposits display unexpected hyperactivity [48, 49], which might represent an early sign of neuronal excitotoxicity associated with impending synaptic failure and incipient cognitive decline [50, 51]. In this respect, the possible protective role of aNPCs in mouse models of AD, where TNFα is increased and neurons display hyperactivity, deserves future investigation.

## Conclusions

We demonstrated that aNPCs revert the TNFα-induced increase in frequency and amplitude of mEPSCs in cortical neurons through the activation of CB1 receptors. By preventing the potentiation of excitatory neurotransmission in cortical neurons, which represents the main target for neurodegeneration in AD and other neurodegenerative pathologies, aNPCs may have significant translational potential.

## Additional file

Additional file 1: Figure S1. AM251 treatment has no effect on synaptic activity, cell viability and CB1 receptor expression. A Representative electrophysiological traces and group data of average mEPSC amplitude and frequency of control (n = 10) and AM 251 treated (n = 9) cells (scale bar: 20 pA, 200 ms). B Fluorescence images of Hoechst and propidium iodide double staining in cortical neurons and summary bar graph showing lack of cytotoxic effects after AM251 treatment (scale bar: 40 μm). C, D Confocal microscopy images and summary bar graph showing expression of CB1 receptors in control and AM251 treated cultured neurons (scale bar: 20 μm). All data are expressed as mean ± standard error of the mean. *CB1* type 1 cannabinoid receptor, *mEPSC* miniature excitatory post-synaptic current. (PPT 1143 kb)

## Abbreviations

AD: Alzheimer’s disease
aNPC: Adult neural stem cell
CB1: Type 1 cannabinoid receptor
CNS: Central nervous system
GFP: Green fluorescent protein
LPS: Lipopolysaccharide
LPS^+^aNPC: Lipopolysaccharide-stimulated adult neural stem cell
mEPSC: Miniature excitatory post-synaptic current
NPC: Neural stem/precursor cell
PI: Propidium iodide
SVZ: Subventricular zone
TNFα: Tumor necrosis factor alpha
