# Supplementary figures and images for: Subventricular zone neural progenitors reverse TNF-alpha effects in cortical neurons
Source: Stem Cell Res Ther. 2015 Sep 7;6(1):166. doi: 10.1186/s13287-015-0158-2 (PMC4562198; doi:10.1186/s13287-015-0158-2)

## Slide 1
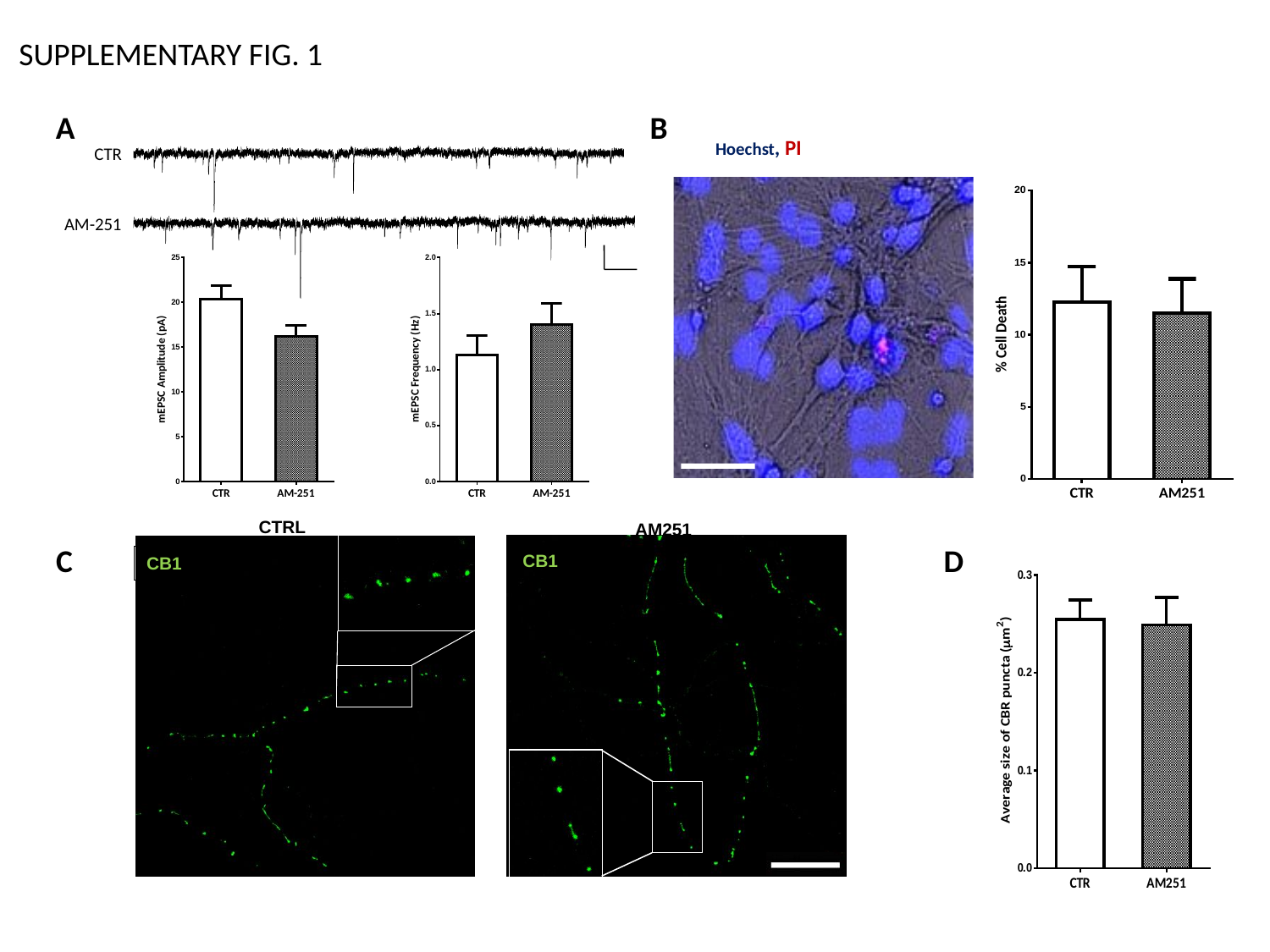

SUPPLEMENTARY FIG. 1
A
B
Hoechst, PI
CTR
AM-251
CTRL
AM251
C
D
CB1
CB1

Supplement: Additional file 1: Figure S1. — AM251 treatment has no effect on synaptic activity, cell viability and CB1 receptor expression. A Representative electrophysiological traces and group data of average mEPSC amplitude and frequency of control (n = 10) and AM 251 treated (n = 9) cells (scale bar: 20 pA, 200 ms). B Fluorescence images of Hoechst and propidium iodide double staining in cortical neurons and summary bar graph showing lack of cytotoxic effects after AM251 treatment (scale bar: 40 μm). C, D Confocal microscopy images and summary bar graph showing expression of CB1 receptors in control and AM251 treated cultured neurons (scale bar: 20 μm). All data are expressed as mean ± standard error of the mean. CB1 type 1 cannabinoid receptor, mEPSC miniature excitatory post-synaptic current. (PPT 1143 kb) [file 13287_2015_158_MOESM1_ESM.ppt]
